# SGLT2 inhibition improves coronary flow velocity reserve and contractility: role of glucagon signaling

**DOI:** 10.1186/s12933-024-02491-w

**Published:** 2024-11-15

**Authors:** Sven O. Göpel, Damilola Adingupu, Jue Wang, Elizaveta Semenova, Margareta Behrendt, Rasmus Jansson-Löfmark, Christine Ahlström, Ann-Cathrine Jönsson-Rylander, V. Sashi Gopaul, Russell Esterline, Li-Ming Gan, Rui-Ping Xiao

**Affiliations:** 1 Global Patient Safety BioPharmaceuticals, AstraZeneca, Gothenburg, Sweden; 2 Research and Early Development, Cardiovascular, Renal and Metabolism (CVRM), BioPharmaceuticals R&D, AstraZeneca, Gothenburg, Sweden; 3 College of Future Technology, Peking University, Beijing, 100871 China; 4 Data Sciences and Quantitative Biology, Discovery Sciences, R&D, AstraZeneca, Cambridge, UK; 5 Late CVRM, Biopharmaceuticals R&D, AstraZeneca, Gaithersburg, USA; 6 Ribocure Pharmaceuticals AB, Gothenburg, Sweden & SuZhou Ribo Life Science Co. Ltd., Gothenburg, Sweden; 7 Drug Metabolism and Pharmacokinetics, Research and Early Development, Cardiovascular, Renal and Metabolism (CVRM), BioPharmaceuticals R&D, AstraZeneca, Gothenburg, Sweden; 8 Department of Cardiology, Department of Molecular and Clinical Medicine, Institute of Medicine, Sahlgrenska Academy at the University of Gothenburg, Gothenburg, Sweden; 9 Imperial College London, School of Public Health, Department of Epidemiology and Biostatistics, London, United Kingdom

**Keywords:** Metabolic syndrome, Heart failure, SGLT2 inhibitor, Coronary flow velocity reserve, Cardiac contractility, Glucagon, Argenine/ADMA ratio, Echocardiography

## Abstract

**Background:**

SGLT2 inhibitors, a T2DM medication to lower blood glucose, markedly improve cardiovascular outcomes but the underlying mechanism(s) are not fully understood. SGLT2i’s produce a unique metabolic pattern by lowering blood glucose without increasing insulin while increasing ketone body and glucagon levels and reducing body weight. We tested if glucagon signaling contributes to SGLT2i induced improvement in CV function.

**Methods:**

Cardiac contractility and coronary flow velocity reserve (CFVR) were monitored in ob/ob mice and rhesus monkeys with metabolic syndrome using echocardiography. Metabolic status was characterized by measuring blood ketone levels, glucose tolerance during glucose challenge and Arg and ADMA levels were measured. Baysian models were developed to analyse the data.

**Results:**

Dapagliflozin improved CFVR and contractility, co-application of a glucagon receptor inhibitor (GcgRi) blunted the effect on CFVR but not contractility. Dapagliflozin increased the Arg/ADMA ratio and ketone levels and co-treatment with GcgRi blunted only the Dapagliflozin induced increase in Arg/ADMA ratio but not ketone levels.

**Conclusions:**

Since GcgRi co-treatment only reduced the Arg/ADMA increase we hypothesize that dapagliflozin via a glucagon-signaling dependent pathway improves vascular function through the NO-signaling pathway leading to improved vascular function. Increase in ketone levels might be a contributing factor in SGLT2i induced contractility increase and does not require glucagon signaling.

**Graphical Abstract:**

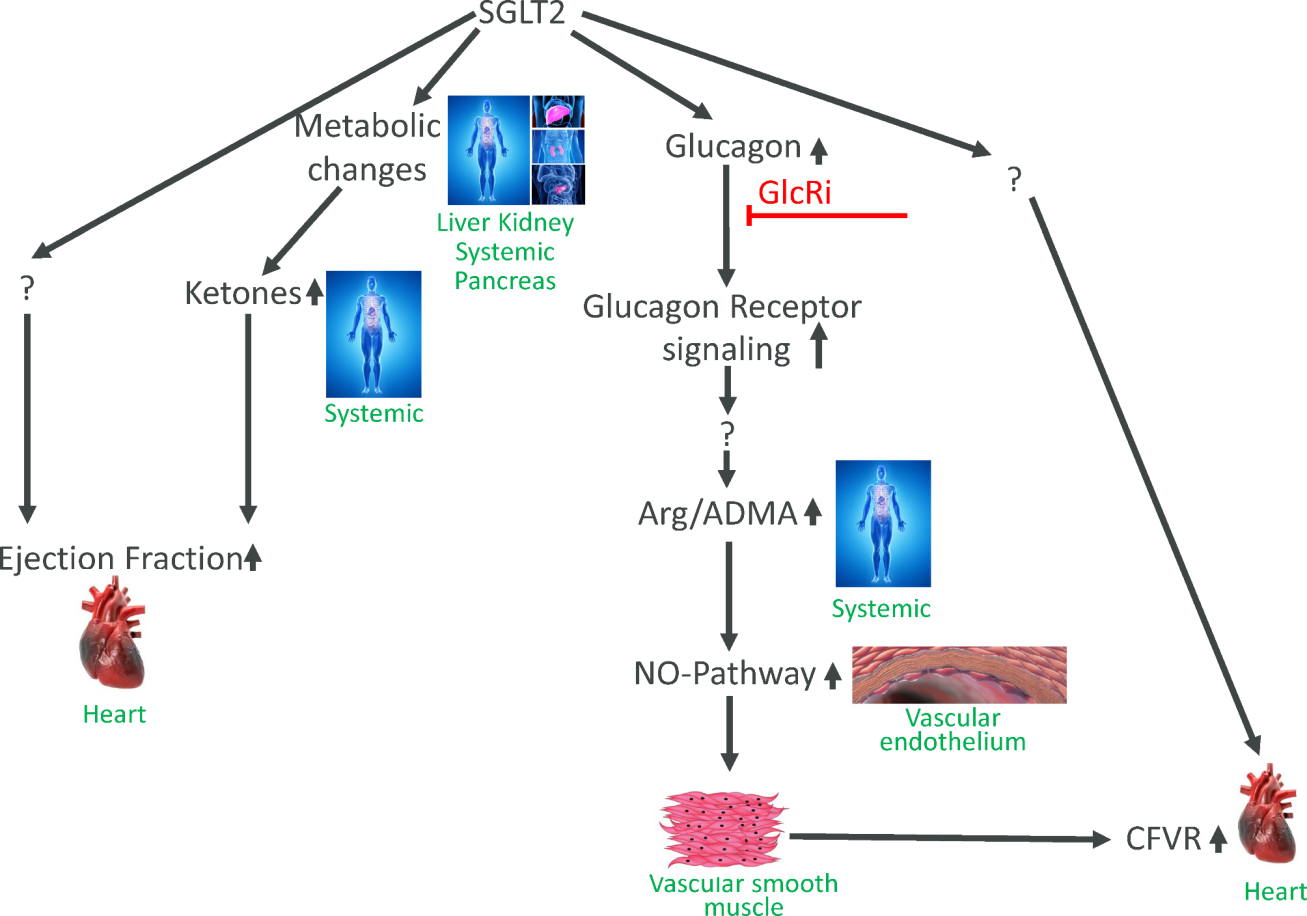

**Supplementary Information:**

The online version contains supplementary material available at 10.1186/s12933-024-02491-w.

## Background

Type 2 diabetes mellitus is one of the major risk factors for heart failure and it has been recognized that both micro- and macrovascular complication occur in T2DM patients leading to an increased risk of CV events. In numerous clinical trials it has been shown that SGLT2i’s substantially reduce the risk of death and hospitalization due to heart failure [1–3] and increases quality of life [4, 5]. These beneficial effects are not solely related to the blood sugar lowering effect since other anti-diabetic treatments with even larger blood glucose lowering effect do not result in the pronounced improvement in cardiovascular outcome. In addition, SGLT2i’s improve CV outcome irrespective of the diabetic state of the heart failure patients [2, 6] Multiple potential mechanisms [7] mediating the positive effects of SGLT2i on CV function have been proposed and understanding the underlying mechanisms is crucial for patient segmentation and the development of future improved treatments.

Leptin-signaling deficient mice recapitulate the major metabolic responses to SGLT2 inhibition found in clinic studies including the lowering of HbA1c, reduction in body weight, increase in ketones and the increase in glucagon/insulin ratio [8–10]. In addition, ob/ob mice are also a model for heart failure and we have previously shown that treating ob/ob mice with empagliflozin improves left ventricular contractility and coronary flow velocity reserve (CVFR) [11]. Importantly, ob/ob mice do not develop atherosclerosis and changes in CFVR thus reflect microvascular events [12]. Both empagliflozin and dapagliflozin are highly selective inhibitors for SGLT2 over SGLT1. SGLT2 expression is considered to be kidney restricted. Whilst some publications have suggested extra-renal expression in rat aorta and porcine left arterial circumflex endothelial cells [13, 14] compelling evidence remains to be established. Consequently we believe the CV effects we report here are likely the result of metabolic and hormonal balance changes. SGLT2i’s are unique in their overall catabolic profile: (1) Blood glucose is lowered mainly by direct urinary excretion and NOT via an increase in insulin levels. (2) As a consequence of the lowered blood glucose levels, plasma insulin levels drop. (3) SGLT2 inhibition produces a net caloric loss due to glucose leaving the body (4) SGLT2 inhibition modestly increases the levels of ketones both in clinical and preclinical studies and finally (5) SGLT2i increase the blood glucagon levels which is also a major catabolic signal. Based on our previous findings that SGLT2i treatment of ob/ob mice increased the Arg/ADMA ratio, which can be viewed as an indicator for endothelial NO-pathway activity since arginine is the substrate and ADMA an inhibitor for NO synthase [15], we hypothesized that this might be a pathway by which SGLT2i’s improve CFVR [11]. The improved contractility seen with empagliflozin might be causally linked to increases in β-hydroxybutyrate since administration of ketones has been shown to improve left ventricle contractility in T2D patients [16] and ketones have been linked to improved CV function in both humans and several animal models [17].

Glucagon is a major metabolic hormone and its main function is to mobilize glucose from the liver via glycogenolysis or gluconeogenesis to protect against potentially life-threatening hypoglycemia. Inhibition of glucagon receptors has been proposed as anti-diabetic treatment and glucagon receptor inhibitors (GcgRi) have even been tested in clinical trials confirming their blood glucose lowering action [18, 19]. Glucagon has been claimed to be pro inotropic and chronotropic [20–22] in human and has been proposed as treatment for β-blocker and Ca^2+^-channel inhibitor overdose, resistant cardiac failure and myocardial infarction (see [23] for review). The ino- and chronotropic effects are only seen at unphysiologically high concentrations, much higher than the SGLT2i induced glucagon levels and therefore cannot explain any of the beneficial CV effects seen with these drugs in recent outcome trials. In human, it has also been shown that glucagon increases lactate extraction by the heart from the blood which could indicate improved oxygen supply since metabolization of lactate requires oxygen. Interestingly, this effect of glucagon is only seen in patients suffering from coronary artery disease, however dose dependence of this effect has not been established [21]. In addition, in the perfused rat heart glucagon within the physiological range promoted activation of PI3 kinase in cardiac tissues similar to insulin [24]. This lead to improved glucose utilization whereas the inotropic effects only appeared at very high concentrations. Taken together, these effects could be seen as indications for reduced basal oxygen demand potentially resulting in lowered basal coronary flow since coronary flow and oxygen demand are tightly linked. The reduced basal flow could then contribute to the increased coronary flow reserve. It is however questionable to what extent direct effects of glucagon on the myocardium translate to human (for review see [25]) and we show in our current studies evidence for glucagon signaling dependent systemic elevation of the Arg/ADMA ratio which potentially explains improvement in vascular function not requiring direct action on the myocardium.

Based on the above and the fact that SGLT2 inhibitors increase glucagon levels [26, 27], a feature that to our knowledge is not shared by any other blood glucose lowering drug, we investigated in our current studies the contribution of glucagon signaling to improvements in CV function. For this we used dapagliflozin in combination with a GcgRi in 2 preclinical in vivo studies. First, using dapagliflozin we confirmed our main findings on metabolism and CV-function from our previous empagliflozin ob/ob mice study showing that SGLT2 inhibition produces a shift to a more catabolic metabolic state and has 2 distinct effects on cardiac function (1) improved CFVR and (2) improved left ventricular contractility. Second, we show that cotreatment with GcgRi blunts the dapagliflozin-induced improvement in CFVR whereas increased ventricular contractility is unaffected by GcgRi cotreatment. Third, we extend our main findings obtained in rodents to non-human primates. Rhesus monkey with metabolic syndrome respond to dapagliflozin treatment with similar metabolic changes and exhibit improved CFVR and left ventricular contractility. As in the rodent study, the dapagliflozin-induced improvement on CFVR was sensitive to GcgRi co-treatment, whereas the effects on ventricular contractility appear to be independent from glucagon signaling.

## Methods

### Mouse study

Methods and procedures were previously published in [11]. Animal care and experiments comply to the Swedish national regulations L150 for the protection of animals used for scientific purposes. The project protocol was assessed and approved by the Gothenburg Regional Animal Ethics Committee for Animal Experimental permit no 86 2015. In short homozygous male C57BL/6 J-lepob mice [ob/ob−/− mice] (Jackson Laboratory, USA) were randomized into control (*n* = 18), low (1.5 mg/kg, *n* = 19) and high dose of dapagliflozin (4.0 mg/kg, *n* = 18) and combo treatment group (dapagliflozin 4.0 mg/kg + glucagon receptor inhibitor 20 mg/kg, *n* = 18). Dapagliflozin was given in drinking water and glucagon receptor inhibitor was mixed in R3 diet. Blood samples were collected from the tail veins 1 week prior to each ultrasound scanning for HbA1c determination at about 7 to 8 (baseline), 15 to 16 (5 weeks after intervention) and 20 to 21 (10 weeks after intervention) weeks of age, under non-fasting conditions. At termination, blood was taken for Arg, ADMA and ketones in the vena saphena after 4 h fasting in the awake mouse. LC–MS/MS analysis of L‑arginine and asymmetric dimethyl arginine (ADMA) was analyzed using a modified liquid chromatography–tandem isotope dilution mass spectrometry analysis (LC–MS) methodology previously described. β-hydroxybutyrate (nmol/well) was measured using colorimetric probe with an absorbance band at 450 nm (Abcam, USA).

Cardiac echocardiography and CFVR was measured using high‑resolution ultrasound imaging Transthoracic echocardiography using non-invasive Doppler ultrasound at 9, 16 and 21 weeks of age in all mice using a high-frequency ultrasound imaging system with a 40-MHz central frequency transducer to characterize coronary vascular function according to previously established protocols [11, 12]. Mice were anesthetized by inhalation of isoflurane gas. All flow velocities were determined from signals that were stable for at least three consecutive heartbeats and representative of the average of two cardiac cycles. Measurements was performed offline in a blinded fashion. Measurements of left ventricle dimensions were performed in standard B-mode using images in short axis views at the papillary level, before the CFVR protocol. FAC was calculated as 100 * ((LV diastolic area– LV systolic area)/LV diastolic area), where LV is the left ventricle.

### NHP study

The present PK and efficacy studies were approved by the Institutional Animal Care and Use Committee of Peking University. The housing and experimental procedures were in accordance with the principles of laboratory animal care of the National Academy of Sciences/National Research Council. The main and efficacy study was performed using 29 male Rhesus monkeys of metabolic syndrome as characterized in the previous study [28] at the AAALAC International accredited animal facility at Peking University. Animal housing: individual cages, 12-hour light and dark cycle, temperature range 18–24° and humidity of 40–70%. The animals had free access to water and were ad libitum fed with national standard pellet monkey chow (Beijing HFK Bio-Technology Co. Ltd.), containing 7–10% crude fat, 16–20% crude protein, and 55–65% crude carbohydrate. Animals were randomized into the either vehicle (*N* = 10), dapagliflozin (*N* = 9, 0.975 mg/kg) or Combo treatment (*N* = 10, dapagliflozin 0.975 mg/kg + the GcgRi adomeglivant 2.15 mg/kg), for eight weeks. Additionally, blood samples from the efficacy study were collected after eight weeks and analyzed for both dapagliflozin and adomeglivant concentrations in the combination study to ensure exposure of both drugs.

Blood samples were taken from the saphenous vein after 14–16 h of fasting and anesthesia with ketamine (10 mg/kg, iv., Jiangsu Zhongmu Beikang Pharmaceutical Co., Ltd). The biochemical assays were performed using Roche 4000 and e411 analyzer series. Intravenous glucose tolerance test (IVGTT) was performed as described previously [28].

Transthoracic echocardiography was performed using a high-resolution transducer (Vivid 7 dimension, 5 S transducer, GE Vingmed Ultrasound) with electrocardiogram (ECG) and blood pressure monitored during the measurement. CFR was assessed by echocardiography in a modified apical view of the distal part of left anterior descending (LAD) coronary artery. After baseline recording, adenosine (380 ug/kg/min) was administered by intravenous infusion, and hyperemic flow were recorded. CFR was calculated as the ratio of hyperemic to baseline peak diastolic coronary flow velocity. Dobutamine stress echocardiography (DSE) was conducted afterwards when the coronary blood flow, blood pressure and HR was back to the basal level. Dobutamine (Shenyang Everbright Pharmaceutical Co., Ltd) was administered by intravenous infusion at the doses of 30ug/kg/min and 60ug/kg/min.

### Statistics

We have applied Bayesian multilevel statistical models to analyze most of the data. Multilevel models [29] allow modelling of data measured on different levels at the same time, thus taking complex dependency structures into account. The Bayesian framework combines prior knowledge (if available) and the observed data to arrive at the posterior distribution of a quantity of interest. Such approach provides a natural way to express uncertainty and derive probability statements for every quantity of interest. Multiple comparison adjustments, widely discussed in the literature as one of main flaws of traditional (“frequentist”) statistics [30, 31], is not required for Bayesian models as all information about a parameter or outcome is contained in its posterior. We have used the *brms* [32, 33]R-package to implement the models. The number of Bayesian models applied in a variety of fields is rapidly growing, including drug development and pharmaceutical research [34, 35]. All data are presented as mean +/- SEM.

## Results

### Study lay out and analysis

**Fig. 1:**
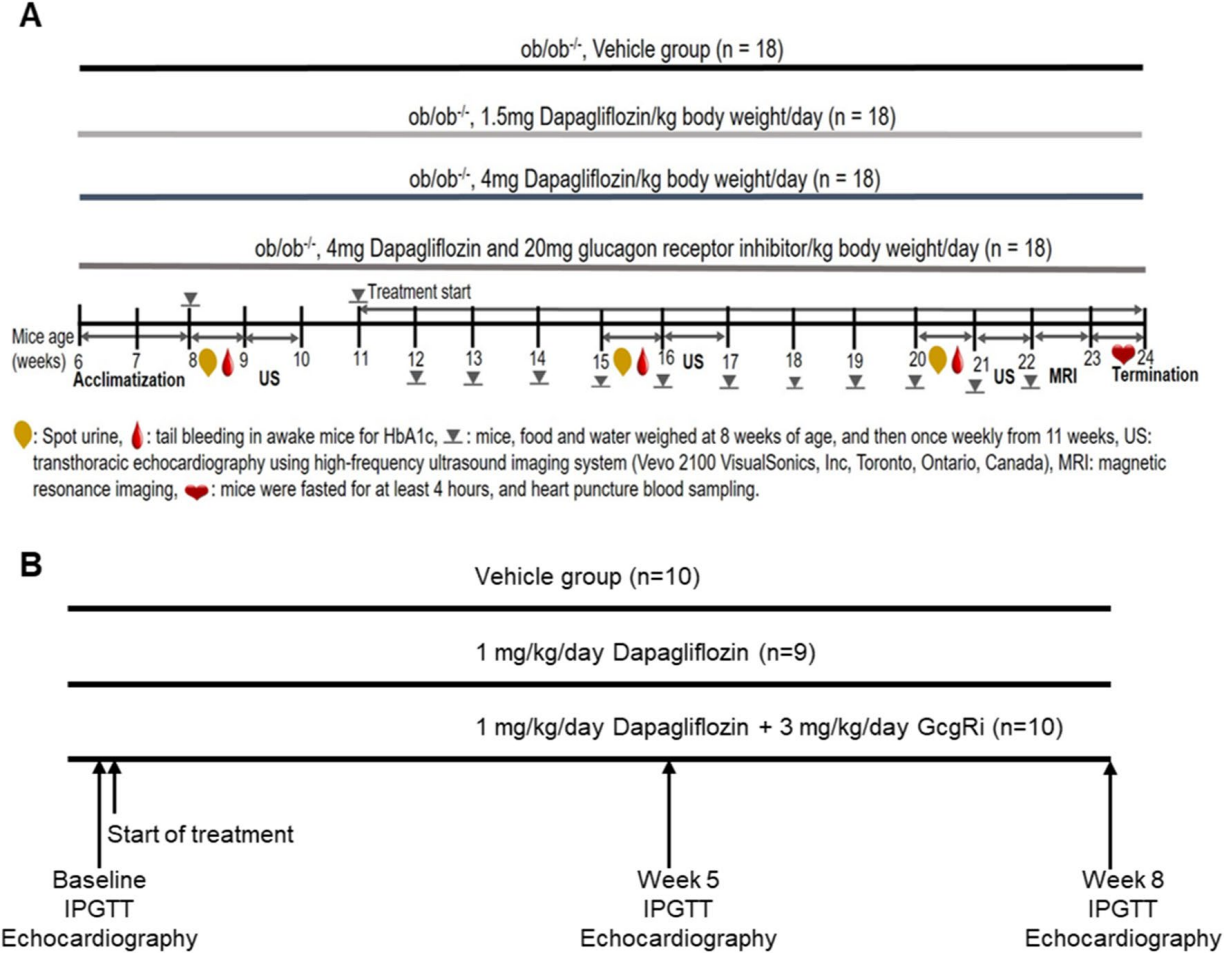
Study layout. The figure depicts the study layouts for the ob/ob mice (**A**) and metabolic syndrome rhesus monkey study (**B**). 18 mice were randomized into the 4 treatment groups and blood samples drawn and ultrasound (US) performed after as indicated. For the rodent study 29 animals were randomized into the vehicle (*n* = 10), Dapagliflozin (*n* = 9) and Combo group (*n* = 10). IPGTT and echocardiograph were performed as indicated

Ob/ob mice were randomized into 4 groups and treated with either vehicle (contr), low dose Dapa (LD), high dose Dapa (HD) or high dose Dapa in combination with a glucagon receptor inhibitor (HD + GcgRi). For the non-human primate study (NHP), rhesus monkey with metabolic syndrome were randomized into 3 groups and treated with vehicle (contr.), dapagliflozin (D) or combination of dapagliflozin and the GcgRi (D + GcgRi). The study layouts are depicted in Fig. 1. Most of the data were analyzed using Bayesian statistics and values for evidence ratio and posterior probability for the indicated hypotheses are given. The evidence ratio is an indicator for how much more likely it is that a hypothesis is true than not and can have values from 0 to infinity where 1 means that it is equally probable that the hypothesis is true or not true.

### Dapagliflozin induces a catabolic switch in ob/ob mice

**Fig. 2:**
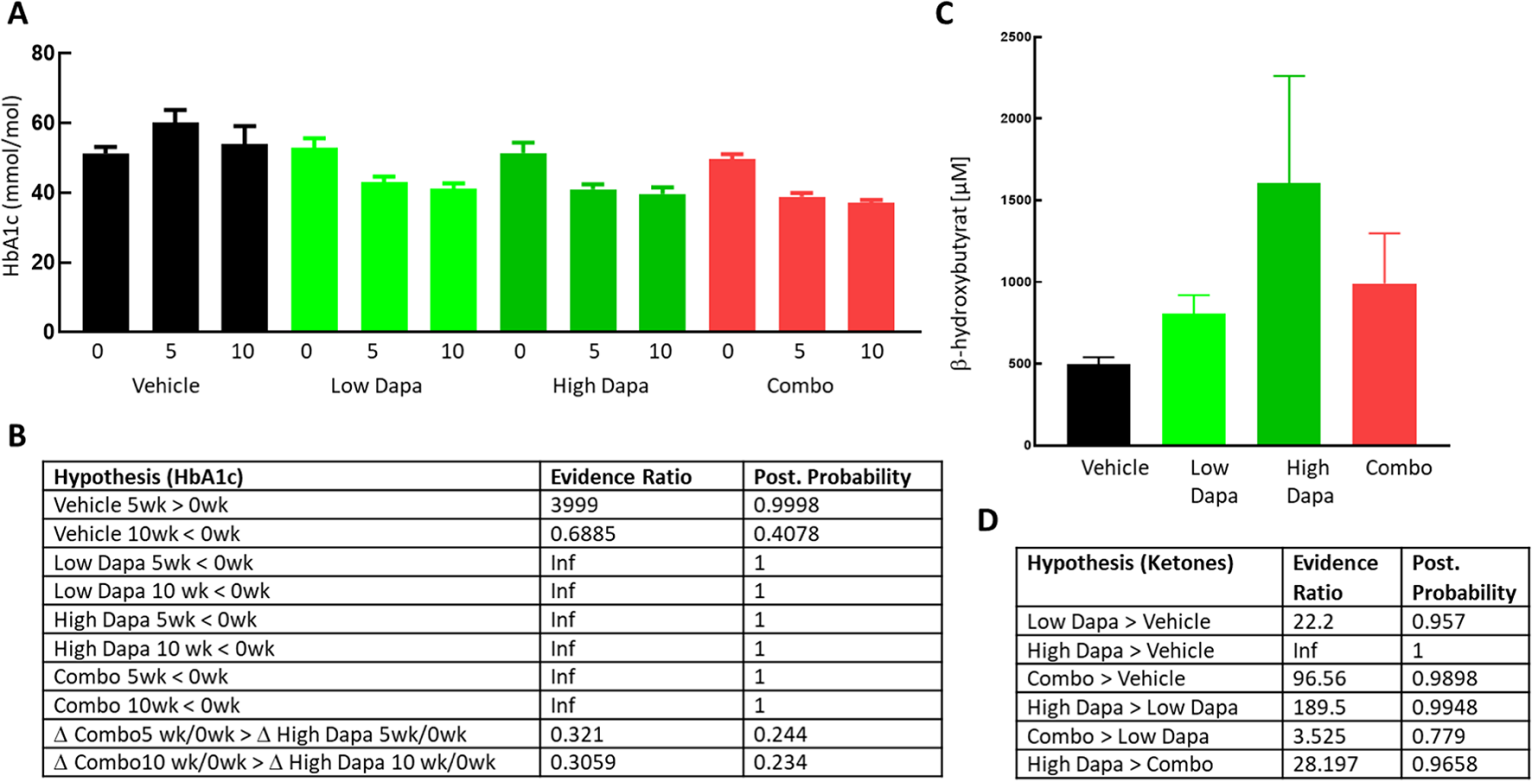
Glucose control and ketone levels in mice. HbA1c levels in ob/ob mice at base line and after 5 and 10 weeks treatment (**A**) for the treatment groups (*n* = 18 in each group) as indicated. Values for each individual animal are indicated as symbols together with average +/- SEM for each group at each time point. Evidence ratio and posterior probability based on Bayesian statistics for the indicated hypothesis for the HbA1c (**B**) and ketone data (**D**) The 2 lower lines in (**B**) show the parameters for the hypothesis that the change in HbA1c from baseline after 5 weeks Combo treatment (**D** Combo5 wk/0wk) is larger than the change for HbA1c during high Dapa treatment (**D** High Dapa 5wk/0wk) and analog after 10 weeks in the last row. (**C**): β-hydroxybutyrate (average +/- SEM) plasma concentrations at termination for the 4 different treatment groups as indicated

In accordance with our recent [11] and other studies in ob/ob mice [8] SGLT2 inhibition clearly lowers HbA1c in all rodent treatment groups (Fig. 2A–B) with evidence ratios reaching infinity (Fig. 2B) in both dapagliflozin and the combo groups. All treatments lowered HbA1c after 5 weeks of treatment compared to the baseline value for each group.

At termination, mean β-hydroxybutyrate levels (Fig. 2C) where higher in all treatment groups, 497 +/- 42.3, 806 +/- 113.5, 1605 +/- 657 and 990 +/- 308 µM for vehicle, LD, HD and HD + GcgRi respectively, evidence ratios and posterior probability are given in Fig. 2D. In the low dapagliflozin group there was a modest increase in β-hydroxybutyrate with an evidence ratio of 22, meaning that it is 22 times more likely that ketone levels are elevated compared to the vehicle group than not. The increase in ketones was clearly dose dependent since the evidence ratio reached infinity when comparing the HD with the vehicle group and evidence ratio for the hypothesis that ketones in the high dapagliflozin group were higher than in the low dose group reached 190. Surprisingly (see discussion) adding the GcgRi on top of the high dose dapagliflozin gave only a weak reduction in ketone levels. The mean β-hydroxybutyrate concentration in the combo group was between that in the low and high dose dapagliflozin groups and the evidence ratio for ketones being higher in the combo group compared to vehicle was 96.6.

### Catabolic switch in NHP

**Fig. 3:**
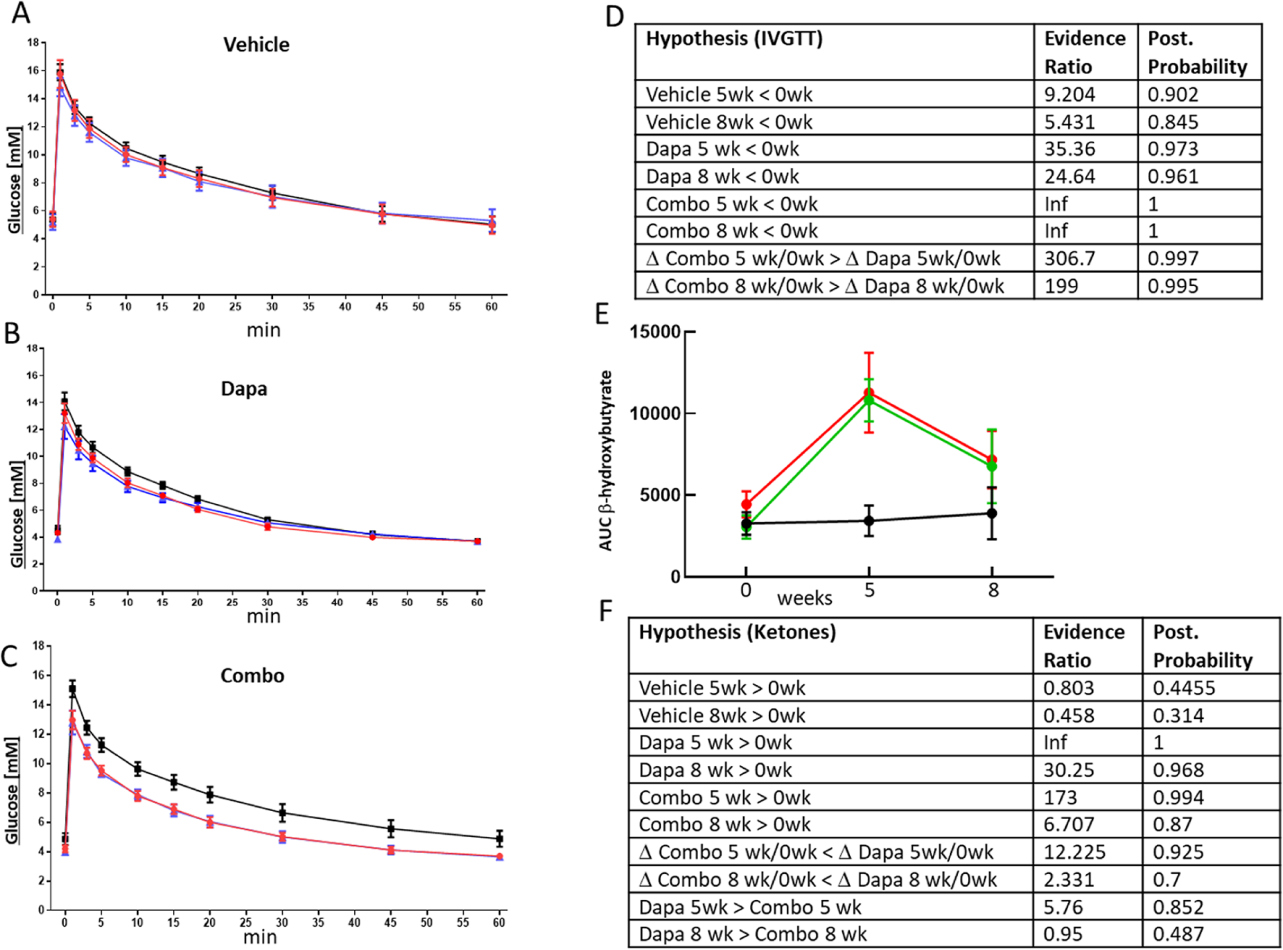
Glucose tolerance and ketone levels in NHP. Glucose concentrations during IVGTT in NHP for vehicle (**A**), dapagliflozin (**B**) and combo (**C**) treated animals. For each treatment group glucose concentrations at baseline (black) and after 5 (blue) and 8 weeks (red) treatment are depicted. AUC for the data shown in **A**–**C** were calculated (not shown) and analyzed using Bayesian statistics. Resulting evidence ratio and posterior probability Bayesian statistics for the indicated hypothesis are shown in (**D**). AUC’s for ketone levels obtained from same samples as used for **A**–**C** are shown in **E** in black for vehicle, green for Dapa and red for combo treatment. Results from Bayesian analysis with accompanying hypothesizes are summarized in (**F**)

We attempted to test if the effects seen with dapagliflozin and the GcgRi in rodents translated to primates for which we used a NHP study layout similar to the rodent study (Fig. 1B). The IVGTT revealed that glucose tolerance was improved in both treatment groups (Fig. 3B and C) whereas glucose tolerance was fairly stable in the vehicle group throughout the study (Fig. 3A). From the data depicted in Fig. 3A–C, AUC’s were calculated and analyzed using Bayesian statistics. Evidence ratios and posterior probabilities are shown in Fig. 3D. There was a tendency for AUC’s to decrease during the study in the vehicle group. Evidence ratios for AUC being smaller at 5 and 8 weeks compared to baseline in the vehicle group were 9.2 and 5.4 respectively. However, the decline in AUC’s in the treatment groups was clearly higher with higher evidence ratios (35 and 24 for dapagliflozin and infinity in the combo group). Importantly, the decline in AUC in the combo group was more pronounced compared to the dapagliflozin only group (Δ Combo5 wk/0wk > Δ Dapa 5wk/0wk) which is a clear sign for target engagement of hepatic glucagon receptors leading to improved glucose tolerance (throughout the text Δ indicates difference between the indicated conditions e.g. Δ Combo5 wk/0wk means difference between value obtained after 0 and 5 weeks Combo treatment).

SGLT2 inhibition increased β-hydroxybutyrate levels (Fig. 3E) expressed as AUC during the IVGTT in NHP. Evidence ratios for dapagliflozin only and combo treatment raising ketones compared to baseline after 5 weeks treatment were infinity and 173 for dapagliflozin and combo treatment, respectively, but only 0.8 for the vehicle group (Fig. 3F). Similar to the mice study, Co-treatment with GcgRi did not affect ketone levels much compared to dapagliflozin alone and AUC’s in the dapagliflozin and combination treatment groups were similar with evidence ratios for ketones being higher in the dapagliflozin group than in the combo group at only 5.76 and 0.95 at 5 and 8 weeks respectively. In addition, when comparing the increase in the 2 treatment groups after 5 and 8 weeks, evidence ratios for a larger increase in the dapagliflozin group compared to the combo groups reached only 12.2 and 2.3. The outcomes are similar to the mice study with glucagon receptor inhibition having only a small impact, if any, on ketone levels.

### Coronary flow velocity reserve in mice

**Fig. 4:**
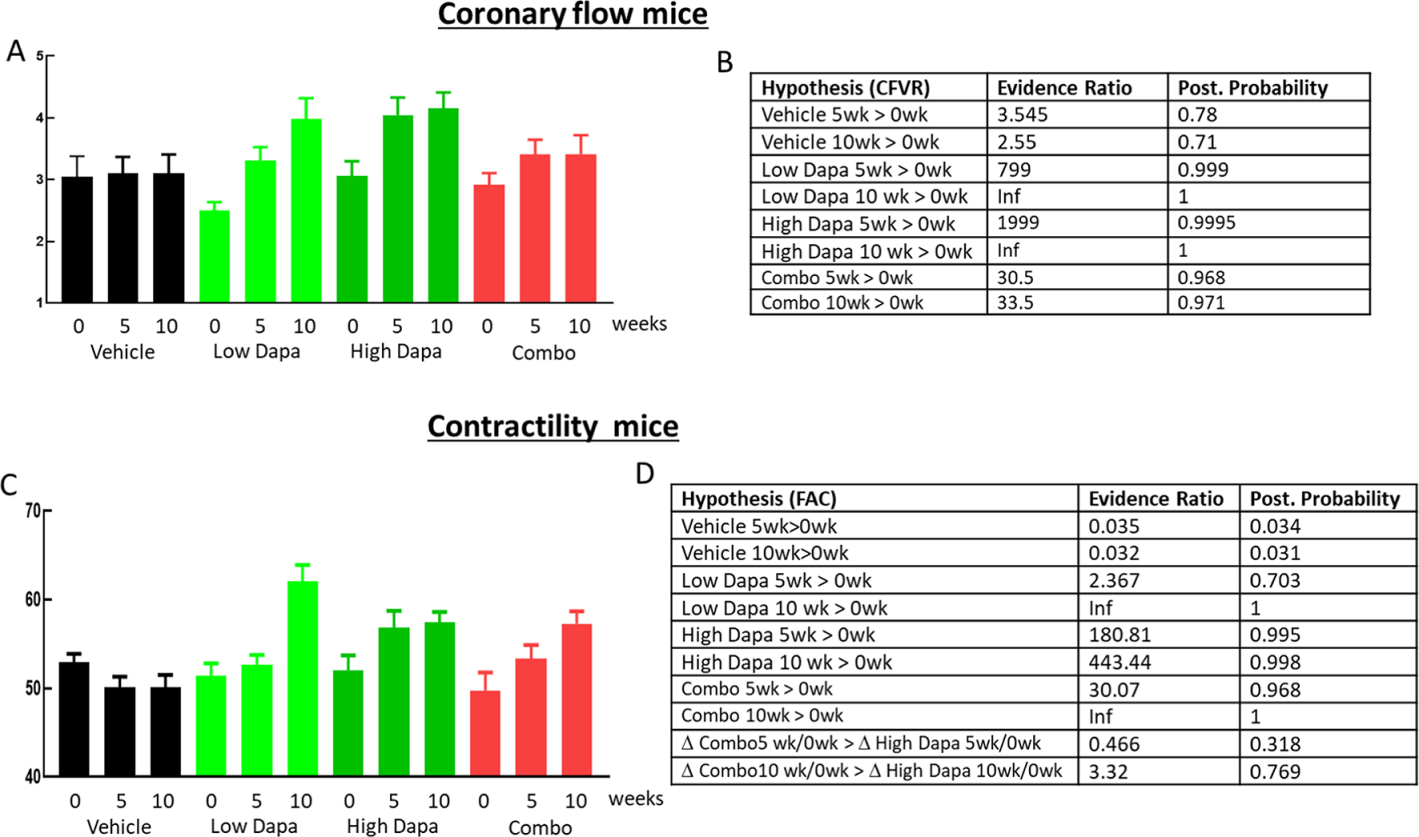
Cardiovascular function in ob/ob mice. Coronary flow velocities were measured as described under methods and coronary flow velocity reserves calculated by dividing stimulated with basal flow velocity. Flow reserves are shown (**A**) for baseline and after 5 and 10 weeks treatment as indicated. Data are color coded for the treatment groups vehicle (black), low dapagliflozin (light green), high dapagliflozin (dark green) and combo treatment (red). Data are shown as average +/- SEM. Contractility expressed as fractional area change (FAC) are shown in **C** for the same time points and color coding for treatment group as in (**A**). Evidence ratios and posterior probabilities based on Bayesian statistics for the indicated hypothesis are given in (**B**) for CFVR and in (**D**) for contractility

In order to assess the impact of dapagliflozin on cardiac function and its dependence on glucagon signaling, we performed echocardiography before and during acute 2.5% isoflurane stress with resulting CFVR values shown in Fig. 4A and the outcome of the Bayesian analysis in Fig. 4B. In the vehicle group, CFVR was stable with evidence ratios for CFVR after 5 and 10 weeks being higher compared to baseline at 3.55 and 2.55. Similar to our previous study [11], in both dapagliflozin groups there was a clear increase in CFVR with high evidence ratios and posterior probability (Fig. 4B) for CFVR being higher after 5 and 10 weeks compared to baseline. Importantly, adding the GcgRi on top of the high dapagliflozin dose almost completely blunted the improvement in CFVR with the evidence ratio at 10 weeks dropping from infinity and 1999 for the dapagliflozin only groups to 33.5 in the combo group. This shows that dapagliflozin-induced improvement in CFVR is at least partially glucagon receptor signaling dependent.

### Coronary flow in NHP

**Fig. 5:**
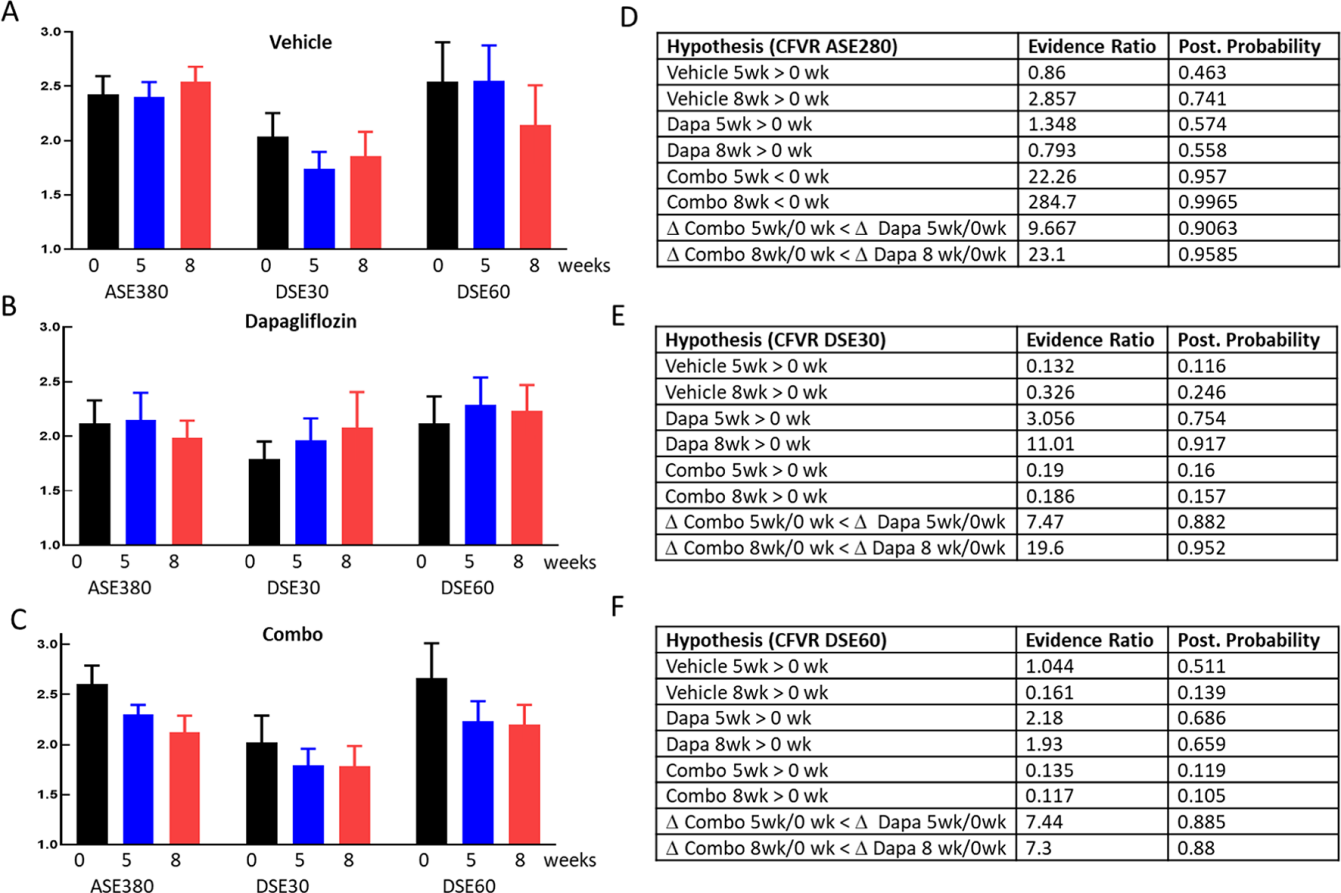
Coronary flow in NHP. Coronary flow velocity reserve for the vehicle (**A**), dapagliflozin treated (**B**) and combo treated (**C**) are shown as average +/- SEM. CFVR was calculated by dividing adenosine (ASE280), low dobutamine (DSE30) and high dobutamine (DSE60) stimulated flow velocity by the basal flow. For each condition and stressor CFVR was measured at baseline (black) and after 5 (blue) and 8 (red) weeks of treatment as indicated. Results from Bayesian statistics analysis together with the hypothesis are shown for adenosine (**D**), low dobutamine (**E**) and high dobutamine (**F**) stimulation

Since the NHP model for CFVR is less well established, we measured coronary flow during basal condition and during acute stimulation with adenosine (ASE), low (DSE30) and high dose (DSE60) of the β1-agonist dobutamine. Adenosine stimulated CFVR was stable throughout the study in the vehicle and the Dapagliflozin group (Fig. 5A and B left) and declined in the combo group (Fig. 5C left) with evidence ratios for CFVR being smaller after 5 and 8 weeks of treatment reaching 22 and 284 respectively (Fig. 5D). Comparing the changes (Δ) in CFVR between the dapagliflozin only and the combo group yields evidence ratios of 9.7 and 23 (5 and 8 weeks) with the combo group experiencing a larger decline in CFVR than the dapagliflozin group (Fig. 5D). This indicates that the combo treatment diminished the adenosine responsiveness of CFVR whereas dapagliflozin did not affect adenosine stimulated CFVR. Next, we analyzed the CFVR using low and high dose dobutamine stress (DSE30 and DSE60). In all groups, the high dose dobutamine produced a larger increase in CFVR compared to the low dose showing that maximal flow is not achieved with the lower dose. In the vehicle group there was no consistent change of dobutamine-induced CFVR during the study. In the dapagliflozin group there was a time dependent increase in CFVR during low dobutamine stress with evidence ratios reaching 3.1 and 11 after 5 and 8 weeks (Fig. 5E). Also, when comparing the changes of CFVR during the study between the combo and the dapagliflozin group, the evidence ratio for the ΔCFVR was larger in the dapagliflozin group compared to the combo group showing 7.5 and 19.6 (Fig. 5E) at 5 and 8 weeks, respectively. During high dose dobutamine stimulation, dapagliflozin treatment had no effect, possibly because maximal flow was already achieved with the high dose leaving no room for additional effect with dapagliflozin. However, there was still a modest effect with evidence ratio at 7.4 and 7.3 when comparing ΔCFVR between dapagliflozin and combo treatment (Fig. 5F). In summary, dapagliflozin improved CFVR during low dose dobutamine stress and this improvement was blunted when adding the glucagon receptor antagonist on top of dobutamine. In addition, the combo treatment lowered adenosine induced increase in CFVR.

### Cardiac contractility in mice

In mice, contractility was measured as FAC (fractional area change) before the CFVR protocol during 1% isoflurane anesthesia. FAC (Fig. 4C) improved after 5 weeks of high dose dapagliflozin treatment and after 10 weeks in all three treatment groups with evidence ratios reaching infinity, 443 and infinity for low dose, high dose and the combo group respectively after 10 weeks (Fig. 4D). In the untreated animals, FAC did not increase throughout the study. Opposite to dapagliflozin’s effect on CFVR, its effect on contractility was not glucagon signaling dependent since adding the GcgRi did not affect dapagliflozin-induced improvement in contractility.

### Cardiac contractility in NHP

**Fig. 6:**
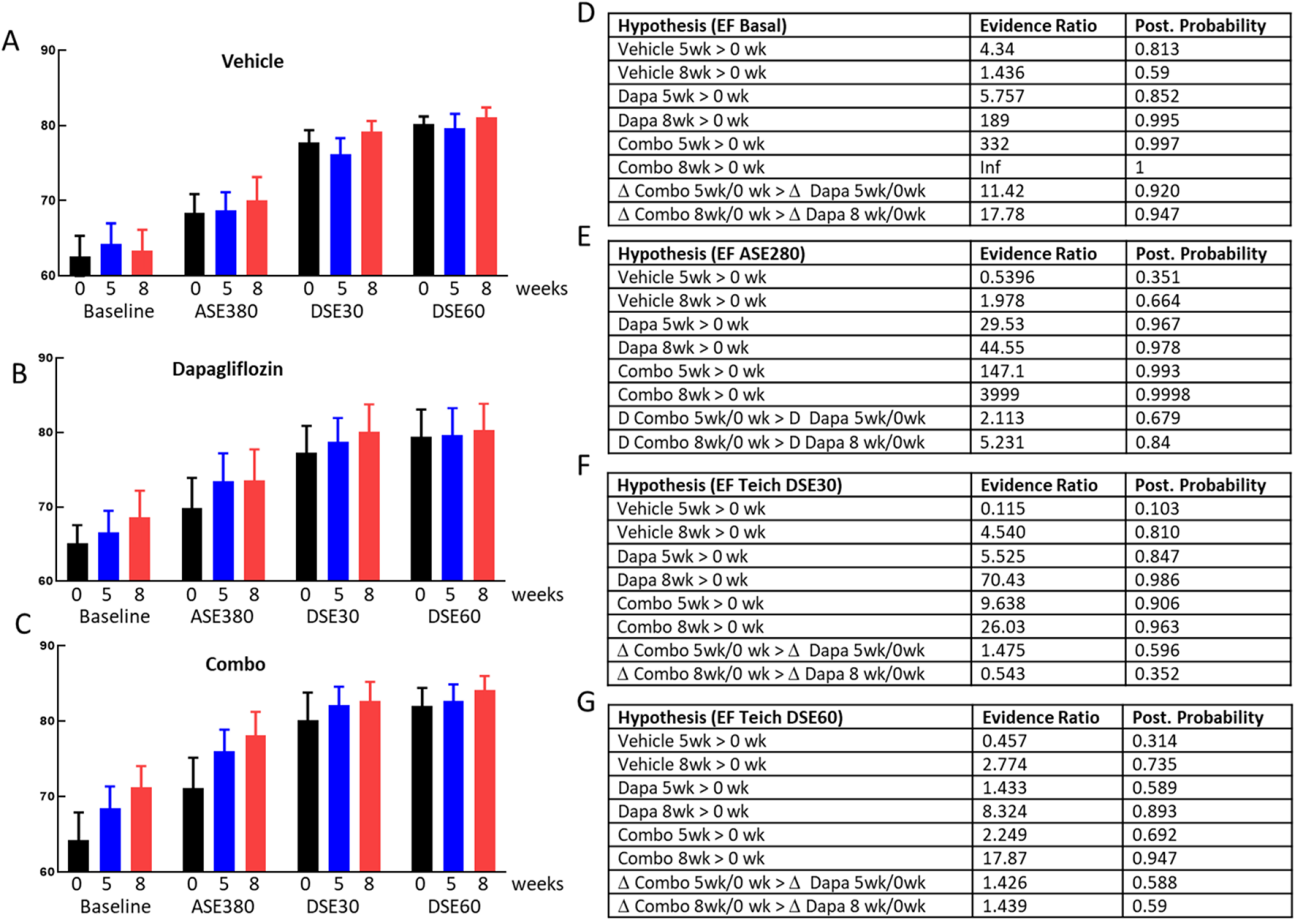
Contractility in NHP. Ejection fraction averages +/- SEM for the vehicle (**A**), dapagliflozin (**B**) and combo treated animals are shown without stimulation (Baseline) and after adenosine, low (DSE30) and high (DSE60) dose dobutamine stress. Contractility was measured before (0 weeks, black) and after 5 (blue) and 8 (red) weeks of treatment with either vehicle, dapagliflozin or combo

Since methods for measuring SGLT2i effect on CV function is considerably less well established in NHP we measured contractility at each time point before stress and during adenosine and 2 doses of dobutamine stress, as for the CFVR measurements. At all timepoints for all groups contractility measured as ejection fraction (EF) was the lowest at basal conditions, increased during adenosine infusion and further enhanced during dobutamine infusion (Fig. 6A-C). Similar to rodents, contractility was stable throughout the entire study in the vehicle group (Fig. 6A) with all evidence ratios lower than 4.6 for any change in the vehicle group (Fig. 6D-G). In the dapagliflozin and combo treatment group there was a time dependent increase in contractility during basal and adenosine stimulation with high evidence ratios for all treatments improving contractility (Fig. 6D-E). During low and high dobutamine stimulation, treatment effects were still present both in the dapagliflozin and combo groups but were much less pronounced compared to adenosine and basal conditions. Evidence ratios for both treatments producing higher contractility compared to pretreatment during low dose dobutamine stress reached 5.5 and 70.4 for dapagliflozin at 5 and 8 weeks and 9.6 and 26 in the combo group at the same time points (Fig. 6E). During high dose dobutamine stress, data were qualitatively similar to the low dobutamine dose data with a time dependent increase of EF in both treatment groups, albeit less pronounced compared to what was observed during basal, adenosine and low dose dobutamine conditions. Analogous to the data on CFVR, we attribute the smaller treatment effect observed during high dose dobutamine compared to low dose dobutamine to the fact that high dose dobutamine is a strong stressor already causing maximal effect on cardiac function making it difficult to observe an additional dapagliflozin treatment effect. In summary, we observed similar responses in the NHP compared to the rodent: dapagliflozin improves contractility and this improvement was not affected by glucagon receptor inhibition.

### Arg/ADMA ratio

**Fig. 7:**
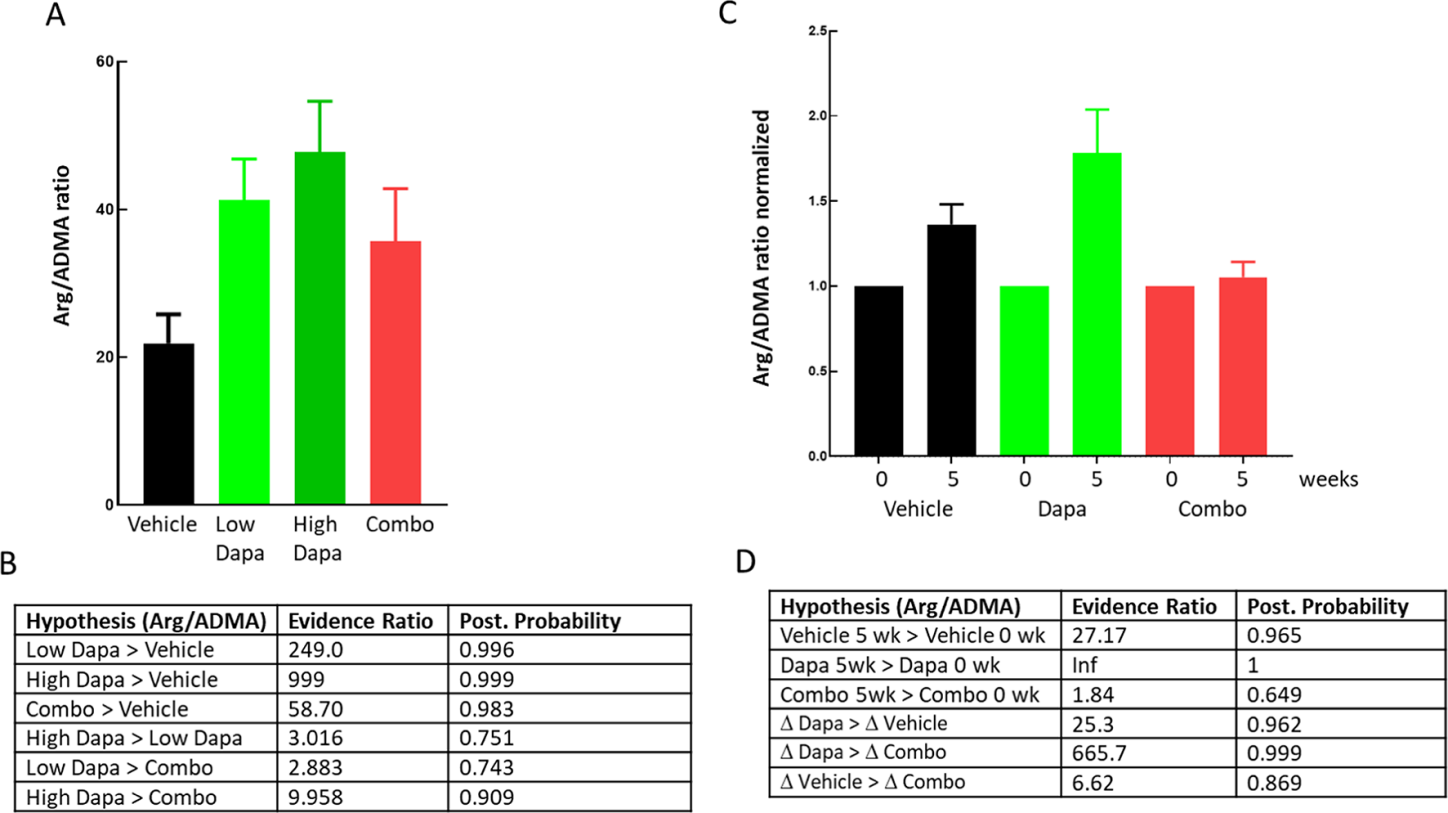
Arg ADMA ratios in mice and NHP. Arg/ADMA ratios from blood samples taken at termination in ob/ob mice (**A**) as indicated. Normalized Arg/ADMA ratios in NHP (**C**). Arg/ADMA ratios were determined in blood samples taken before and after 5 weeks of treatment in all animals. Values obtained at 5 weeks were normalized to the baseline measure. Results from Bayesian statistics analysis together with the hypothesis are shown for mice (**B**) and NHP (**D**)

In the mouse study, L-arginine and ADMA were measured at termination after a 4 h fast (Fig. 7A). In both dapagliflozin groups, the Arg/ADMA ratio was increased with an evidence ratio of 249 and 999 for the treated animals having a higher ratio compared to the vehicle group (Fig. 7B). The Evidence ratio for the Arg/ADMA ratio being larger in the combo group than in the vehicle group was 58.7. Evidence ratios for the low and high dapagliflozin groups being higher than the combo group was 2.8 and 10. In the NHP study Arg/ADMA was measured both before onset of treatment and after 5 weeks of treatment, giving more reliable data and stronger statistics compared to the rodent study (Fig. 7C). The evidence ratios for Arg/ADMA being higher after 5 weeks (compared to baseline) was 27.2, infinity and 1.8 for the vehicle, dapagliflozin and combo group respectively (Fig. 7D) showing that the dapagliflozin induced increase in Arg/ADMA ratio was glucagon signaling dependent in both species. The lower sensitivity of Arg/ADMA ratio to glucagon receptor inhibition in the rodent study might be due to larger spread of the data and lack of repeated measurements compared to the NHP study.

## Discussion

Ob/ob mice develop insulin resistance due to leptin deficiency, become severe obese with moderate hyperglycemia and high insulin release capacity [36] and are a model for metabolic syndrome/pre-diabetes [37]. They do develop coronary microvascular dysfunction but not macrovascular defects [12], thus any changes in CFVR must reflect microvascular changes. In addition, they exhibit cardiac contractile dysfunction [38] which we have previously shown may serve as a useful model for SGLT2 inhibitor effects on CV function and metabolism [11]. Changes in CFVR in human are a composite of micro and macrovascular effects, therefore findings from rodent studies regarding CFVR may differ from human. To translate our findings into primates we used rhesus monkeys with metabolic syndrome. These animals are not diabetic and have only weak cardiovascular dysfunction compared to the ob/ob mice and functional changes in response to treatment are thus expected to be less pronounced. The Bayesian statistics used in this assessment have several advantages: (1) The strength of statistical signal is not affected by multiple comparisons. (2) More complex statistical models can be developed making it possible to test several hypothesis simultaneously, independent of each other. (3) Bayesian statistics calculates the probability of a hypothesis being true which is what one actually is interested in. The casual perception of the *p*-value as posterior probability of the truth of the null hypothesis is false [39]. Furthermore, *p*-values are routinely dichotomised based on an arbitrary threshold of 0.05. Hence, we avoid this approach in our analysis and rely on the strength of Bayesian modelling.

Dapagliflozin improved contractility measured as ejection fraction in both species (Figs. 4 and 6) and the outcome in the current mouse study was similar to our previous ob/ob mouse study using empagliflozin. For the monkeys, contractility was only affected by treatment during basal, adenosine and low dose dobutamine stimulation but not during high dobutamine stress. We attribute the lack of treatment effect during high dobutamine stress to the strong contractility achieved with dobutamine alone. The ~ 80% ejection fraction achieved with high dose dobutamine appears to be at the upper limit in healthy rhesus monkeys [40, 41] and thus, dapagliflozin and combo treatment cannot increase ejection fraction any further during high dobutamine stress. Both in monkey and mice adding the GcgRi on top of dapagliflozin did not reduce contractility showing that SGLT2i induced contractility improvement is glucagon signaling independent.

We hypothesize that the dapagliflozin-induced increase in contractility is at least partly associated with the increase in ketone levels but do not exclude any other potential pathways. Firstly, it has been shown that ketone administration increases contractility in patients suffering from heart failure [16]. In addition, ketones are reduced in patients with heart failure with reduced ejection fraction (HFrEF) compared to both healthy subjects and heart failure with preserved ejection fraction (HFpEF) patients [42]. Secondly, we show here in both rodents and in monkeys that β-hydroxybutyrate levels (Figs. 2 and 3) moved in parallel with effects on contractility (Figs. 4 and 6) with dapagliflozin increasing both contractility and ketone levels and addition of GcgRi not reversing these effects. The finding that inhibition of the glucagon receptor did not reverse the β-hydroxybutyrate increase in response to SGLT2 inhibition was unexpected since it is generally assumed that ketone production is regulated by the insulin/glucagon ratio and that inhibition of GcgR should therefore lower ketone levels. However, in mice it was recently shown that SGLT2 inhibition increases ketone levels independent of glucagon signaling and that inhibition of the GcgR did not affect ketone levels [9], confirming the validity of our findings. Thirdly, we are not aware of any mouse in vivo studies investigating the effect of ketone infusion on cardiac contractility in mice, however in Bdh1 transgenic mice, in which ketones are elevated, the animals were partially protected against contractile dysfunction after transverse aortic constriction [43]. Fourthly, culturing adult rat myocytes in physiological relevant β-hydroxybutyrate concentration range improves sarcomere shortening and Ca^2+^ fluxes [44]. Ketones have been proposed as “super fuel” but recently the potential beneficial effects of ketones on cardiac and kidney function have been extended beyond its properties as energy source and anti-inflammatory effects via NLPR3 inflammasome formation, antioxidative stress via SIRT1 activation, amelioration of hypoxia, reduction of myocardial infarct size [45] and other mechanisms have been suggested (for review see Ekanayake and Mudaliar [46]).In summary in both our mice and NHP studies, dapagliflozin improves left ventricular contractility and, as found in numerous clinical trials with SGLT2i’s, increases ketone levels. Both effects are independent of glucagon receptor signaling. Although we are not excluding any other possible mechanisms, we hypothesize that ketones at least partly mediate the positive effects on contractility after SGLT2i treatment.

As for cardiac contractility, we confirmed our previous rodent data showing that SGLT2 inhibition improves CFVR and we further demonstrate here that this also translates into NHP. In mice this improvement was strongly blunted when co-treating with the GcgRi (Fig. 4). In NHP we stimulated CFVR with adenosine and dobutamine. Overall, treatment effects on CFVR in NHP were much less pronounced compared to results in rodents most likely because the NHP’s used here are much less pathologic then the ob/ob mice used. However, we did find statistical support for the hypothesis that dapagliflozin increased CFVR during acute low dose dobutamine stress whereas no treatment-dependent response was seen during acute adenosine and high dose dobutamine stress. Analogous to the NHP contractility data, this might be explained by the fact that CFVR was close to maximum during acute adenosine and high dobutamine application and therefore dapagliflozin treatment could not elicit any additional CVFR increase. The effect of dapagliflozin on CFVR was clearly glucagon signaling dependent and adding the GcgRi not only blunted the dapagliflozin induced increase at low dose dobutamine stress but also lowered CFVR during acute adenosin and high dose dobutamine stress (Fig. 5). The difference in GcgRi sensitivity shows that SGLT2 inhibition affects contractility and CFVR via different pathways.

The changes in CFVR are paralleled by changes in the Arg/ADMA ratios in both species (Fig. 7). Dapagliflozin increases the Arg/ADMA ratio and the addition of GcgRi either diminishes or completely blunts this increase. Nitric oxide is one of the major vascular dilators and is produced by NO-synthase which uses arginine as a substrate. ADMA is an arginine analog, endogenously produced during degradation of methylated proteins and inhibits NO-synthase [47]. The Arg/ADMA ratio affects coronary flow, is a marker for the NO-pathway and has been shown to be a predictor of CV outcomes [48]. Interestingly, SGLT2i has been shown to increase eNOS activity and NO production [49]and improve endothelial function [50]. Also, adenosine induced increase in myocardial perfusion in human is at least partial dependent on NO increase as shown by Buus et al. 2001 [51]. Based on the above and our data, we hypothesize that SGLT2 inhibition via unknown pathways improves this pathway in a glucagon signaling-dependent fashion, however we do stress that we are not excluding any other additional pathways. SGLT2 inhibition in mice improves NO-dependent coronary artery relaxation in vivo but not ex vivo suggesting that the SGLT2 effects on vascular function are not mediated via direct interference with SGLT2 in cardiovascular tissues [52]. In addition, in a rat model of diabetes SGLT2i treatment improves endothelial dependent but not independent aortic relaxation [53]. These findings support the view that SGLT2i-induced improvement of vascular function is at least partially endothelial dependent and is due to an overall systemic change of metabolism or mediators and not due to direct SGLT2 inhibition in cardiovascular tissue. However, this does not exclude other mechanisms e.g. it has been demonstrated that SGLT2 blockers also inhibit sodium-hydrogen exchanger 1 (NHE-1) in the heart and vascular tissues [54, 55]. Inhibition of NHE-1 by SGLT inhibitors have thus been implied generating positive cardiovascular effects in reperfusion and infarct models, reducing cardiac inflammation, fibrosis and oxidative stress and also improving vasodilation (for review see [56]).

Effects of glucagon on vascular beds have been demonstrated in rat, dogs, NHP and human. Glucagon lowers systemic vascular resistance, glucagon immunoneutralization reduces blood flow to stomach and liver (for review see [57]) but high glucagon doses increase the blood flow to the kidney [23]. Cardiovascular effects of glucagon are well established with both iono and chrontrophic actions. In addition, glucagon infusion in humans leads to increased coronary flow velocity. However this is paralleled by corresponding increase in oxygen demand due to increased contractility, heart rate and cardiac output [58]. Thus, at least for cardiac vascular beds, the effect of glucagon on coronary flow appears to be downstream to the effects on oxygen demand glucagon has. To our knowledge, direct effects of glucagon on vascular cells have not been demonstrated. Taken together the above argues against a direct effect of glucagon on CFVR but rather for an overall indirect systemic effect to which glucagon contributes.

It has been proposed that SGLT2i might have direct effects on cardiac macrovascular beds [13, 14]. Since mice do not develop macrovasular disease the reduced cardiovascular function in ob/ob mice is attributable to microvascular effects. We believe that the effects of dapagliflozin described here are not mediated via a direct effects on SGLT2 in cardiovascular tissues but more likely attributable to metabolic and hormonal balance changes.

Recently it has been proposed that restoring fasting state by SGLT2 inhibition via mTOR deactivation could lead to improved endothelial function [59]. Indeed it has been demonstrated that SGLT2i treatment in mice on high fat diet increased phosphorylation of eNOS and decreased mTOR phosphorylation in the heart [60]. However, we are not aware on any studies offering an explanation for how SGLT2i’s or GcgR inhibition could interfere with the Arg/ADMA ratio.

Beneficial effects with SGLT2i treatment may be even more pronounced in CKD patients than in patients with impaired CV function [61] and there are some analogies between CKD and CV disease: Subjects with renal failure have elevated ADMA levels [62] as do patients with chronic heart failure [63] and hypertrophic cardiomyopathy [64]. Late stage renal disease is associated with abnormal coronary flow reserve independent of obstructive coronary artery disease [65] and, as for CV function, the kidney is an organ that strongly relies on endothelial function. The kidney is of major importance for the regulation of the Arg/ADMA and is a net producer of the amino acid [66]. To our knowledge there are no studies available clarifying if kidney disease progression causes changes in the Arg/ADMA or the other way round hence it is not clear if kidney disease progression with associated Arg/ADMA disturbance causes reduced CFVR or if both kidney disease and CFVR reduction are downstream of a generally declining systemic Arg/ADMA ratio.

The fact that dapagliflozin improves CFVR in a glucagon-dependent fashion might have clinical implications. Many cardiac arrhythmias are related to myocardial ischemia. The SGLT2i-induced reduction in cardiac arrhythmias and sudden cardiac death [67] could be partially explained by improvement in myocardial perfusion. SGLT2i‘s reduce myocardial infarct size in porcine models and in humans [49, 68]which possibly is related to the effects on vascular function reported here. HFpEF is more closely linked to microvascular dysfunction than HFrEF [69]. SGLT2 inhibitors might thus also be beneficial in HFpEF patients for which few, if any, treatment options are available. In the recent EMPEROR-preserved trial it has been shown that SGLT2 inhibition in fact improves CV-outcome in HFpEF patients [70]. These findings combined with our current study could suggest that SGLT2 inhibition via glucagon signaling dependent pathways improves CVFR contributing to improved CV-outcome. Therefore, SGLT2i combination treatments with glucagon agonists might be beneficial. However, it remains to be shown that increasing glucagon signaling in fact also increases CFVR. Due to some similarities between kidney disease and heart failure, increasing glucagon signaling might also be beneficial in chronic kidney disease patients.

### Study limitations

(1) No ex vivo vasoreactivity study was performed to further dissect mechanisms underlying the observed improved coronary vascular function. However, due to ethical reasons, only non-invasive cardiovascular studies can be performed in the non-human primate metabolic syndrome model. In mice, it is highly challenging to isolate coronary arteries for ex vivo functional studies due to technical hurdles, while a surrogate vascular segment will not necessarily reflect the effect on coronary arteries due to distinct molecular mechanisms controlling vasomotor activities. Further, the mechanisms we describe here are dependent on systemic effects and thus could not have been discovered in an ex vivo setting.

(2) Both the rodent and the NHP study do not include healthy control animals because of the following reasons: (A) Aim of these studies was to gain insight via which mechanisms SGLT2i produce improved CV outcome in patients. Almost the entire clinical literature on CV function and SGLT2i compares effects +/- SGLT2 in patients, not in healthy controls. (B) In order to have an “assay window” we must use models in which there is some impairment of cardiovascular function. In particular for contractility/ejection fraction, it has been demonstrated that it is virtually impossible to induce more contractility if there is no impaired contractility to start with. With the same reasoning we choose these diseased animals and not healthy controls to be able to restore impaired coronary artery function. (C) In particular for the ob/ob mice but also partly for the metabolic syndrome monkeys used here the overall phenotype is very different compared to healthy controls making any comparison questionable: ob/ob mices body weight is 2–3 times higher compared to normal mice which has major impact on behavior. Hormone levels, in particular insulin but also glucagon which is of special interest here are very different. CV function in ob/ob mice is strongly affected making any meaningful comparison with healthy lean animals questionable. (D) For the monkey study we used elderly monkeys suffering from metabolic syndrome. Access to these animals is very limited and there is no age matched healthy control population available. (E) Ethical reasons: due to the reasons above we do not believe that including healthy control mice would add much insight and it is thus difficult to justify use of more animals.

(3) We used ejection fraction as a measure for contractility due to lack of enough high quality loops for strain assessment which would have been preferable. However, although myocardial strain is a more precise measure for contractility it has been shown in head to head comparisons that ejection fraction also is a reliable measure for contractility and correlates highly with strain data in the same individual [71].

## Conclusions

Although the positive cardiovascular outcomes with SGLT2i’s are well established, the underlying mechanisms are still incompletely understood. We show here that SGLT2 inhibition has 2 effects on cardiovascular function. Both in metabolic syndrome NHP and ob/ob mice dapagliflozin improves coronary flow velocity reserve and left ventricular contractility. These functional effects are mediated via 2 distinct pathways. Firstly, co-treatment with a GcgRi abolishes the dapagliflozin induced improvement in CFVR. The effects on CFVR reserve are paralleled by changes in the Arg/ADMA ratio and we propose that SGLT2i induced improvement requires glucagon signaling and involves Arg/ADMA and NO signaling pathways. Secondly, effects on cardiac contractility are glucagon signaling independent and possibly involve increases in ketone levels. These results could contribute to finding more optimal treatments for patients suffering from cardiovascular diseases.

## Electronic Supplementary Material

Below is the link to the electronic supplementary material.

Supplementary Material 1

## Data Availability

No datasets were generated or analysed during the current study.

## Abbreviations

ADMA: Asymmetric dimethylarginine
Arg: Argenine
CFR: Coronary
CFVR: Coronary flow velocity reserve
CV: Cardiovascular
HFpEF: Heart failure with preserved ejection fraction
HFrEF: Heart failure with reduced ejection fraction
HR: Heart rate
Gcg: Glucagon
GcgRi: Glucagon receptor inhibitor
HbA1C: Hemoglobin A1c
LAD: Left anterior descending
NHP: None human primate
NO: Nitric oxide
SGLT2: Sodium glucose co-transporter-2
T2DM: Type 2 diabetes mellitus
