# Supplementary material for: SGLT2 inhibition improves coronary flow velocity reserve and contractility: role of glucagon signaling
Source: Cardiovasc Diabetol. 2024 Nov 15;23:408. doi: 10.1186/s12933-024-02491-w (PMC11568596; doi:10.1186/s12933-024-02491-w)
Supplement: Supplementary file 1 — Supplementary Material 1 [file 12933_2024_2491_MOESM1_ESM.docx]

**Supplemental Material**

***Selection of Dose in NHP***

The doses for the study in rhesus monkeys were selected utilizing a mathematical pharmacokinetic-pharmacodynamic (PKPD) model. The structure of the model was developed based on clinical phase 1 pharmacokinetic (PK), glucagon and glucose data reported for adomeglivant^14^ or dapagliflozin ^22^ administered as single components. The combined dapagliflozin and adomeglivant PKPD model was based on the following assumptions (see Fig S1) : 1) adomeglivant inhibits the glucagon receptor via competitive inhibition with glucagon. Activation of the glucagon receptor by glucagon is then decreased, leading to decreased glucagon-mediated glucose release. 2) Binding of adomeglivant to the glucagon receptor increases glucagon production/release via positive feedback. 3) Dapagliflozin stimulates glucose excretion 4). Dapagliflozin stimulates glucagon production/release (schematic construction is shown in Figure S1).

Figure S1


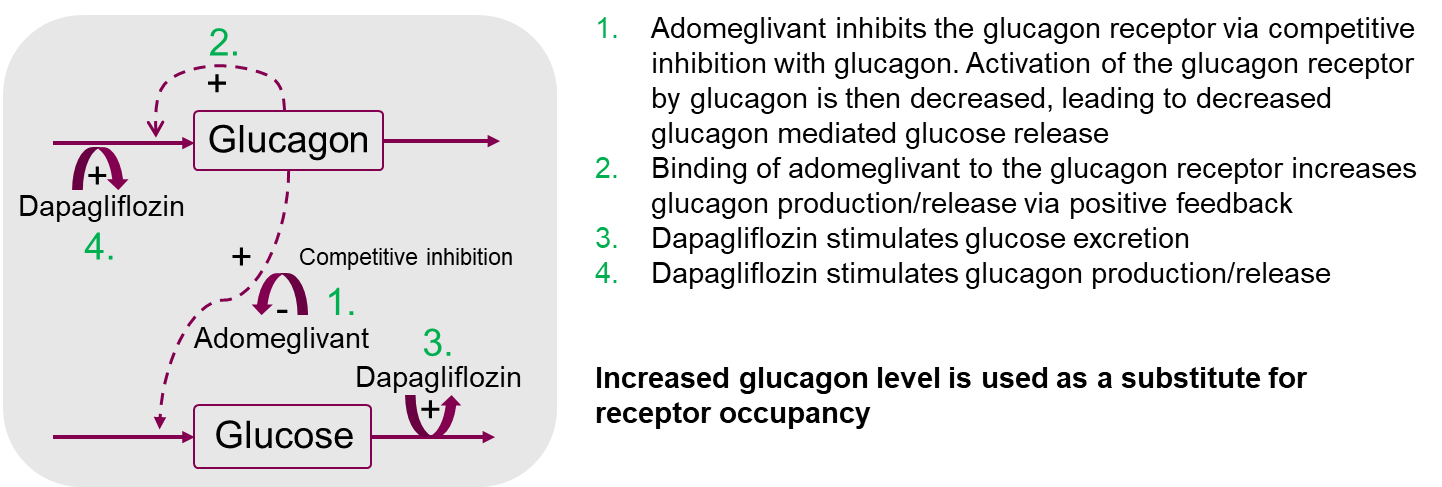


Figure S1: Schematic overview of PK model used to set doses.

Clinical PK data was used throughout the model building process. For the subsequent simulation to NHPs, PK data was generated in rhesus monkeys described below and an NHP PK model was constructed. The NHP PK model was implemented in the PKPD model to enable dose estimation to achieve the desired glucagon elevation.

***Bioanalysis and Pharmacokinetic Analysis to Support Modelling of doses***

A pilot PK study was initially conducted where rhesus monkeys (N=3) were given an oral dose of dapagliflozin (0.5 and 5 mg/kg) and adomeglivant (1 and 5 mg/kg) mixed in orange juice. Blood samples were collected for bioanalysis and PK analysis (C_max_, AUC, t_1/2_, t_max_) at 0, 1, 2, 6, 10, 24h after dosing. Plasma samples were harvested and processed by protein precipitation with acetonitrile containing [^13^C_6_]-dapagliflozin as internal standard (IS) for dapagliflozin and tolbutamide as IS for ademoglivant. Following centrifugation, an aliquot of the organic supernatant was analysed by LC-MS/MS based on reported methods for the simultaneous analysis of dapagliflozin alone or in combination with saxagliptin and metformin in human and rat^55^.

Chromatographic separation of analytes was accomplished on an Ultra performance liquid chromatography (UPLC) system (Waters, UK) connected to an AB Sciex Triple Quad^TM^ 5500 mass spectrometer (Sciex, Canada). The UPLC was equipped with a Waters Acquity UPLC HSS T3 1.8 um (2.1 x50 mm) column with flow rate set at 0.6 ml/min, column temperature at 40°C and injection volume of 2-10 µl. Solvent A consisted of 0.2% formic acid (FA) in water and Solvent B was acetonitrile. The UPLC gradient was as follows: 90% A at 0 min, held for 0.2 min and decreased to 1% at 2.7 min, held for 1 min and reversed to 90% A at 2.9 min and held for 0.1 min. The total run time was 3 min.

The mass spectrometric analysis was conducted in the negative mode and at the following conditions: Curtain gas (40), Collision gas (7), Ion Spray voltage (-4500 V), the Entrance potential (-10), Source Temperature (550 C), Ion Souce Gas 1 (60), Ion Source Gas 2 (70). The Declustering potential (DP), Collision Energy (CE) and Collision Cell Exit Potential (CXP) voltages were optimized for each individual compound and along with the multiple reaction monitoring (MRM) transitions (precursor→product ion) are described in Table S1.

Table S1.

| Precursor  Ion (m/z) | Product Ion (m/z) | | DP | CE | CXP | Compound |
| --- | --- | --- | --- | --- | --- | --- |
| 554 | 218 | -35 | | -44 | -19 | Adomeglivant |
| 453 | 329 | -40 | | -25 | -17 | Dapagliflozin |
| 269 | 170 | -110 | | -23 | -14 | Tolbutamide (IS) |
| 458 | 335 | -40 | | -25 | -17 | [^13^C_6_]-Dapagliflozin (IS) |

DP=declustering potential; CE=collision energy; CXP=collision cell exit potential

Table S1: Source Dependent Settings for adomeglivant and dapagliflozin and their Internal Standards (IS)

**Supplemental Results**

In the PK study, the mean AUC of dapagliflozin at 0.5 mg/kg and 5 mg/kg doses were 317±102 and 3896±1126 ng.h/mL, respectively. The corresponding C_max_ values were 95.3±13.3 and 542±372 ng/mL for the respective doses. The mean AUC of ademoglivant at 1 and 5 mg/kg was 24559±7930 and 189156±113256 ng.h/mL, respectively. The corresponding Cmax values were 881±339 and 4523±3504 ng/mL, at their respective doses.

In the efficacy study, where the animals were given a dose of 0.975 mg/kg of dapagliflozin and 2.15 mg/kg of adomeglivant, the mean plasma concentrations were determined by the same analytical method described above. The mean plasma concentrations of dapagliflozin were 23.7±4.69 ng/ml in the dapagliflozin alone group and 29.6±16.42 ng/mL in the combination of dapagliflozin and adomeglivant group after four weeks of dosing and 22.9±7.2 ng/mL and 29.4±9.24 ng/mL after eight weeks of dosing. This suggests that adomeglivant did not impact the PK of dapagliflozin.
